# Exploring the life experiences of school‐aged children afflicted by tethered spinal cord syndrome: An interpretative qualitative study

**DOI:** 10.1111/hex.13969

**Published:** 2024-01-10

**Authors:** Nan Lin, Mingxian Ren, Yujun Xiang, Jiahuan Li, Dan Wang, Hongzhen Xu

**Affiliations:** ^1^ Nursing Department Children's Hospital, Zhejiang University School of Medicine National Clinical Research Center for Child Health Hangzhou Zhejiang China; ^2^ Department of Paediatrics Zhejiang Taizhou Hospital Wenzhou Medical University Taizhou Zhejiang China

**Keywords:** children, experiences, incontinence, qualitative research, tethered cord syndrome

## Abstract

**Background:**

Children affected by tethered cord syndrome (TCS) encounter multifaceted challenges encompassing educational, familial and social spheres, underscoring the significance of a holistic comprehension of their subjective emotional well‐being and life encounters. Nonetheless, healthcare professionals tend to prioritise the physical functionality of the afflicted individuals throughout the treatment and rehabilitation process, often neglecting the emotional experiences and requirements of these children as they transition into posthospitalization phases.

**Aim:**

To advance the subjective experiences and perceptions of children with TCS upon reintegration into their families, educational institutions and wider societal contexts subsequent to their discharge from medical facilities.

**Methods:**

The study was conducted at the Children's Hospital in Zhejiang. Twelve children aged 8–15 with TCS were included in the study. The research design used an interpretative qualitative approach, utilising semi‐structured interviews as the primary data collection method. Data analysis was performed using reflexive thematic analysis, facilitating a comprehensive exploration of emerging themes and patterns.

**Results:**

Four major themes (and seven subthemes) were identified from the findings: (1) growing pains (a shameful secret, distance between ideal and reality); (2) inappropriate expressions of familial affection (knowing is not understanding, unspeakable guilt); (3) social estrangement (uncomfortable distinctions, familiar stranger) and (4) striving for independence and consistency.

**Conclusions:**

Children affected by TCS exhibit internal sensitivity and challenges in self‐development, family dynamics and social interactions. They aspire to attain a future characterised by independence and freedom, akin to that of their typically developing peers. These findings can help health professionals, families and educators gain a deeper understanding of what it takes to be a child with TCS, and the findings can also serve as a platform for interventions that seek to promote self‐expression in these children so that they can experience life as a meaningful and positive process.

**Patient or Public Contribution:**

This study received support from children with TCS and their guardians during data collection, as well as from the head nurse of the unit. Coresearchers also contributed to design, data collection, analysis and writing.

## 1 INTRODUCTION

Tethered cord syndrome (TCS) encompasses a spectrum of conditions characterised by spinal cord strain, compression and conical hypoplasia. These factors culminate in sensory and motor deficits, deformities in the lower extremities and disruptions in urinary and faecal control.^1^ Aetiologies contributing to TCS pathogenesis include spina bifida and various factors, encompassing genetic, nutritional and environmental influences.^2^, ^3^ Prevalence rates indicate a disproportionate occurrence in developing countries, highlighting the need for more in‐depth preventative healthcare strategies.^4^ TCS significantly impairs children's health‐related quality of life (HRQoL) by presenting profound neurological challenges, including ambulatory difficulties, incontinence and cognitive impairments, thereby impacting their social engagement and educational experiences.^5^, ^6^

Guiding frameworks for understanding the development, disease and life experiences of these children are provided by Bronfenbrenner's Ecological Systems Theory and Erikson's Psychosocial Model. Bronfenbrenner's theory underscores the importance of various environmental layers, ranging from microsystems (family, school) to exosystems (policy, culture), and chronosystems (historical events) in an individual's development.^7^ Studies guided by this theory reveal that the HRQoL of children with chronic diseases or congenital defects is influenced by their family, school and community environments, in addition to their illness severity.^8^, ^9^, ^10^ Erikson's model stresses that individuals encounter various psychosocial crises across life stages that need to be addressed for healthy development.^11^ Applying this model to children with chronic disease, it is evident that physical differences can prompt low self‐esteem during the psychosocial crisis of ‘self‐esteem versus inferiority’, thus hampering normal development.^12^ These theories offer a multifaceted perspective on the experiences of children with chronic illnesses or congenital defects and underline the crucial role of environmental factors and psychological developmental stages in their comprehensive understanding.

The past decade has seen an expansion of literature exploring HRQoL, psychological states and socialisation of children with congenital conditions like TCS. However, the perspective of healthcare providers or parents predominates, with a relative paucity of information regarding children's experiences following their reintegration into family and school postsurgery.^13^, ^14^, ^15^ For devising effective health and social programs, gaining a nuanced understanding of patient experiences is pivotal. During school age, a critical period of cognitive, physical, social and emotional development, children interact more with peers and form complex emotions and self‐concepts.^16^, ^17^ The importance of this stage is magnified for children with congenital defects or disabilities as they grapple with identity formation and increased independence.

Understanding the postsurgery experiences of these children at home and school is crucial for creating tailored support. This study uses qualitative research to explore the challenges faced by children with TCS as they move from the hospital to their daily lives. By doing so, it aims to offer specific strategies to enhance their healthcare and support, potentially improving their overall health and development.

## 2 METHODS

### 2.1 Design

Our research inquiry was situated within the paradigmatic framework of interpretivism and constructivism, which guided our investigation. Within the lens of constructionist epistemology, we recognised meaning and experience as socially constructed and reproduced through the intricate interplay of subjective and intersubjective processes.^18^ Thus, our approach involved interpreting individuals' perceptions and understandings of the world through this particular perspective.

### 2.2 Setting

The study was carried out at Zhejiang Children's Hospital, which serves as a national clinical research centre for child health. This hospital attracts children with TCS from various regions across China, making it a comprehensive and representative setting for conducting the study.

### 2.3 Participants

Eligible children meeting specific criteria were invited to participate in this study. The inclusion criteria encompassed the following aspects: (1) age range of 6–15 years; (2) diagnosed with TCS associated with congenital spinal abnormalities like spina bifida or myelomeningocele; (3) undergoing postoperative rehabilitation phase; (4) discharged from the hospital and returning to school for a minimum duration of 1 month and (5) demonstrating willingness to partake in interviews. Exclusion criteria included (1) assessed cognitive abilities below the age‐appropriate level, as determined by professional evaluation; and (2) the presence of additional physical deformities or impairments. Erikson's psychosocial model, comprising eight stages, prescribes the age parameters for participants in the current study. School‐aged children, per Erikson's construct, are situated at a pivotal developmental intersection, featuring intertwined threads of physical, cognitive, social and emotional growth. It is a period of initiation into formal education and the concomitant necessity of adapting to a widening social milieu, deliberating on personal identity and societal roles and introspecting on individual temperaments, ethical benchmarks and aspirations. Considering the lack of a universal definition for ‘school‐age’, various countries have demarcated it relative to their distinct educational contexts. In alignment with the framework of compulsory education in China, this study identifies school‐age as encompassing children aged 6–15.^19^

### 2.4 Recruitment

The study adopted a purposive sampling technique, selectively including children who, having undergone surgical treatment for TCS and subsequent discharge from the hospital, possessed a substantial understanding and experiential perspective of the research question under consideration.^20^ Parents and children meeting the eligibility criteria were contacted and verbally provided with a comprehensive explanation of the study's objectives. Those expressing interest in participating were then presented with detailed information regarding the broader scope of the study, and interviews were scheduled accordingly to accommodate the participants' availability. To ensure the privacy and confidentiality of participants, explicit assurances were provided, guaranteeing the protection of their personal information. As a token of appreciation for their involvement, participants received a commemorative souvenir as a voluntary incentive to participate in the study. During the recruitment phase, two parents declined their children's participation in this study by withholding consent for discussing sensitive experiences. Consequently, we were unable to enlist these two participants. This decision was motivated by their intent to safeguard their children's privacy and emotional well‐being.

### 2.5 Data collection

This study used a rigorous methodology to conduct semi‐structured, in‐depth, face‐to‐face interviews from February to May 2023. To ensure the well‐being and autonomy of participants, they were explicitly informed of their right to refuse participation, withdraw from the study, terminate the interview or decline to answer any questions at any point. Interviews took place in the waiting room of the outpatient clinic, ensuring privacy and limiting the presence to only the researcher and the interviewee. The duration of the interviews varied between 27 and 61 min, with an average length of 34 min. To maintain consistency and focus, the entire interview process adhered to the predefined interview outline, as outlined in Supporting Information S1: File 1. The interviewer was a woman who is a PhD (N. L.) with training in qualitative research and practical experience in personal interviewing. Building on established relationships formed through daily interactions, the researcher mitigated potential biases stemming from reflexivity in the findings due to the observer effect. All interviews were conducted in Chinese and recorded using encrypted audio equipment, capturing not only verbal but also nonverbal cues, such as facial expressions. Throughout the interviews, the interviewer skilfully used various communication techniques, including encouragement, attentive gaze, probing and strategic use of silence, to elicit detailed and comprehensive information from the participants. Recognising the significance of reflexivity in determining sample size, reflective notes were diligently maintained to address uncertainties encountered during the study. After each interview, interviewers completed reflective notes encompassing emerging themes pertaining to the study objectives, noteworthy consistencies and deviations from prior interviews, and any factors that potentially influenced the interview process.^21^ Through iterative discussions and revisions guided by the study objectives, and upon the identification of no new themes pertaining to the study objectives, the research team made the decision to halt recruitment and transition to the data analysis phase. After each interview, we promptly transcribed and organised the content of the interviews and invited interviewees to verify the transcribed text, and after obtaining their approval and feedback, we kept the information anonymously.

### 2.6 Data analysis

The transcriptions of the interviews were promptly converted into Mandarin text within a 24‐h timeframe. The subsequent analysis of the transcripts was carried out utilising the software tools Word and Excel. A thematic reflective analysis employing an inductive approach was used to examine the interview data. Following the step‐by‐step procedure proposed by Braun and Clarke,^22^ our analytical process involved (1) familiarisation with the data; (2) generating initial codes; (3) generating themes; (4) reviewing potential themes; (5) defining and naming themes; and (6) producing the report. To ensure a comprehensive and nuanced interpretation of the data, our analytical approach was collaborative and reflective, drawing upon the diverse perspectives and experiences of the study's participants. Initially, one researcher (N. L.) meticulously read through the transcripts and manually assigned descriptive codes by identifying similar phrases or words within the narratives. Subsequently, two researchers (N. L. and M. R.) independently developed and reviewed the initial themes, engaging in subsequent discussions and revisions with other members of the research team to finalise and assign appropriate names to the identified themes. The credibility of the data was upheld through various means. First, the inclusion of participants with diverse symptomatology added depth and richness to the study. Second, the investigator's extensive experience in researching and providing care for children with TCS lent valuable expertise to the analysis process. Furthermore, independent coding by the investigator was carried out meticulously, ensuring a thorough examination of the data. Rigorous discussions and scrutiny of the findings and coding further enhanced the reliability of the results. Finally, achieving consensus among the research team on the outcomes solidified the credibility of the data, affirming the robustness of the study's conclusions.

### 2.7 Ethical considerations

This research endeavour obtained the official approval of the Ethics Committee of the Children's Hospital, Zhejiang University School of Medicine, under the designated identifier 2023‐IRB‐0133‐P‐01. The study adhered to the ethical principles outlined in the Declaration of Helsinki. As mandated by the Ethics Committee, subjects aged 8 years and above were required to provide informed consent, accompanied by their parents' endorsement. In this regard, a qualified psychologist was engaged to assess the cognitive development level of the children, ensuring their capacity to provide informed consent aligned with their age. The investigator, before conducting the interview, duly apprised both the children and their parents of the potential advantages and risks associated with the study, emphasising the study's independent nature, unrelated to disease treatment or prognosis. Thorough information pertaining to the study was comprehensively conveyed to both the children and their parents, who subsequently furnished written informed consent as a prerequisite to participation.

## 3 RESULTS

The study cohort comprised a total of 12 children, with five males and seven females. The participants had a median age of 12 years, and a detailed overview of their demographic characteristics can be found in Table 1. The analysis of the gathered data revealed four prominent thematic categories: growing pains, inappropriate expressions of familial affection, social estrangement and striving for independence and consistency. These themes shed light on the experiences and perspectives observed within the studied population.

**Table 1:** Characteristics of the children participating in the study.

ID number	Gender	Age (years)	Grade	Preoperative symptoms	Postoperative symptoms	Duration of attending school (months)^a^
1	Female	14	8	Daytime enuresis, abnormal spinal curvature.	Urine leakage every 2 days.	3
2	Female	9	3	Daytime enuresis, faecal incontinence.	Urine leakage 1–2 times a week.	3
3	Female	10	4	Nocturnal enuresis, gait disturbance.	Occasional foot pain.	2
4	Male	13	6	Urinary incontinence, faecal incontinence.	Urine leakage every 2–3 days, occasional stool leakage.	3
5	Female	12	5	Daytime enuresis, faecal incontinence.	Urine leakage every 2 days.	3
6	Male	10	3	Nocturnal enuresis, abnormal spinal curvature.	Urine leakage 1–2 times a month.	1
7	Male	11	4	Urinary incontinence, gait disturbance.	Occasional foot pain, walking on tiptoe.	3
8	Female	8	2	Daytime enuresis.	Urine leakage 1–2 times a month.	2
9	Female	13	7	Urine leakage after exercise.	Leakage symptom relief.	3
10	Male	15	9	Nocturnal enuresis, faecal incontinence.	Urine leakage one time a week, occasional stool leakage.	1
11	Female	13	7	Nocturnal enuresis.	Urine leakage 1–2 times a month.	2
12	Male	12	5	Urinary and faecal incontinence, gait disturbance.	Urine leakage every 2–3 days, occasional foot pain.	2

^a^ The duration of attending school since discharge from the hospital.

### 3.1 Theme 1: Growing pains

#### 3.1.1 Subtheme 1: A shameful secret

Certain challenges encountered by children with TCS, including incontinence resulting from diminished neurological function, might persist unabated following surgical intervention, potentially enduring over their lifetime. The unpleasant smell of faeces, frequent trips to the toilet and restricted diet made them unhappy, and they believed they had a shameful secret and lived with the constant threat of being exposed as dirty and disgusting.

Mom promised me things would be better after the surgery, but I'm still having some accidents. It's not as bad as it was, but I'm super scared that I might not be able to control my pee in the future, or even something scarier might happen. I'm really unsure about asking my friends from school to come over and play, afraid they might find out about my secret. (P5, 12‐year‐old girl)

This whole thing was so embarrassing and humiliating that I didn't want to tell anyone. It's not like other people could really understand what I'm going through or how I feel, so I figured it was best to just keep it my secret. (P12, 12‐year‐old boy)

Some children chose not to reveal the extent of their distress to friends, family or even parents, viewing incontinence as a deeply personal matter. They believed that disclosing it might make things worse rather than better. Their stories described the anxiety, stress and exhaustion that came with keeping this aspect of themselves hidden.

A few of my classmates caught me wetting my pants a while back, but they never asked me about it. I was so scared they'd find out my secret and tell others. I couldn't bring myself to tell my mom and I felt like I needed to talk to someone about it, but I didn't know who to turn to (starts crying). (P8, 8‐year‐old girl)

Many children experienced negative emotions, such as low self‐esteem, due to their lack of bowel control, unpredictable incontinence and irregular symptoms. These challenges heightened their anxiety and fear, making it daunting for them to venture beyond their homes.

I'm so scared, what if I wet the bed in the school dormitory in the future? I bet people would make fun of me. Incontinence is terrifying, it makes me feel so anxious. But then the anxiety just makes the incontinence even worse… (P2, 9‐year‐old girl)

I was always afraid that people would get close and smell something off about me. Having to go to the bathroom all the time or having to change my clothes really got to me. I think if people really knew what I deal with every day, they'd be grossed out. (P4, 13‐year‐old boy)

#### 3.1.2 Subtheme 2: Distance between ideal and reality

Owing to their medical conditions, a multitude of children found themselves compelled to abandon previously cherished hobbies, with a particular emphasis on certain physical activities that presented a higher risk of injury or necessitated proficient skills.

I'm crazy about basketball, but for now, I'm just stuck watching my best friends have all the fun. I'm uncertain if I'll even get a chance to join them and play in the future. (P6, 10‐year‐old boy)

I used to be part of the dance team at school, but ever since I got sick, I can't go to practice or perform anymore. I mean, how can I do what I love when even regular day‐to‐day stuff makes me so uncomfortable? (P3, 10‐year‐old girl)

The older children showed more concern about their future, expressing worries about education, careers and job opportunities. Some started thinking about their future jobs but felt uncertain about their ability to pursue their desired careers. What they once dreamed of now feels like an illusion fading away, highlighting a huge gap between their physical limitations and their aspirations.

I always dreamed of being a cop, nabbing the bad guys, and helping people. But with my health like it is, I'm not sure if I'll ever get to make that dream come true. (P7, 11‐year‐old boy)

I'm such a sports nut. When I watch the Olympics, I can't help but imagine myself being one of those athletes, just killing it out there … I know it's probably a long shot and my mom even says I'd probably be better off as a writer. (P9, 13‐year‐old girl)

### 3.2 Theme 2: Inappropriate expressions of familial affection

#### 3.2.1 Subtheme 1: Knowing is not understanding

Some children shared that their parents might unintentionally attribute blame, especially when clothing or bedding is soiled due to incontinence. They suspect these reactions might stem from parental expectations for a complete recovery postsurgery or from parental exhaustion, affecting their ability to provide comprehensive care.

Mom always asks me to tell her if I've gone pee‐pee, but I swear, I do it just right each time, yet somehow, some still gets on my pants. She thinks I get so wrapped up in playing that I forget to go potty and end up wetting my pants. It's totally not fair! (P8, 8‐year‐old girl)

Furthermore, these participants are sensing that their parents' heightened attention and disciplinary measures might unknowingly add to their stress. This feeling deepens their reluctance to openly discuss their emotional states, leading to an emotional disconnect from their caregivers.

Every night before hitting the sack, Mom would always ask if I'd made a trip to the bathroom. Each morning, the first thing she'd do is check if I'd had a bed‐wetting mishap. I knew she cared, but it also piled a load of pressure on me. I was worried that if I had an accident like before my operation, Mom would have to clean up my sheets again and I'd catch some heat from her. (P11, 13‐year‐old girl)

Every time I got home from school, my dad would always ask if I was still having leaks. It really got under my skin, and I'd always dismissively reply that I wasn't. Felt like I was living under my parents’ microscope every day. Even though I'm all good now, they still check my laundry for any signs of wet trousers, and it's super stressful. (P7, 11‐year‐old boy)

#### 3.2.2 Subtheme 2: Unspeakable guilt

During interviews, children shared that they often communicate their academic stress or emotional impact due to their health conditions to their parents. Sometimes, this leads to conflicts arising from what they see as excessive parental concern, fostering feelings of guilt and regret.

I don't really want to chat with Mom about all the school stuff, ‘cause she already has a lot on her hands just looking after me and I don't wanna make her worry. But sometimes, I just can't help it and I end up shouting at her—it's like I'm some sort of wild, emotional monster or something. (P1, 14‐year‐old girl)

Other participants indicated that their parents allocate disproportionately more resources—including time and financial investment—towards their own care compared to their healthy siblings. They perceived this as resulting in their parents' delayed work and insufficient attention dedicated to their siblings, leading to a sense of self‐blame among these participants.

Mom kinda loves on me more and spends more time with me than my little bros and sis. They always say that Mom's playing favorites, and every time I hear them whining about it, it kinda makes me feel crummy inside. (P5, 12‐year‐old girl)

Mom can't even go to work ‘cause she's always busy looking after me. She's not only hauling me to the doctor's, but she also gets all worried that I'm way too stressed and drags me to a psychiatrist. And she never ever thinks about getting stuff just for herself. (P11, 13‐year‐old girl)

### 3.3 Theme 3: Social estrangement

#### 3.3.1 Subtheme 1: An uncomfortable distinction

Through the children's narratives, it became apparent that they grappled with experiences that deviated from those of their peers, hindering their quest for normalisation. In interactions with peers and at school, many spoke of instances where leakage problems led to withdrawing socially. This resulted in a feeling of being unable to blend in due to embarrassment. Factors like visible leakage, odours, needing aids or longer restroom routines stood out as reasons for feeling excluded and different in school.

Whenever school planned some fun stuff, my buddies didn't ask me to join them. I know they were worried about my health, but it made me feel super different, like I could never fit back in with our crew again. (P6, 10‐year‐old boy)

I'm not really into the stuff our school plans. Like, say they plan a field trip and I've gotta bunk with my friends, I'd be super scared I'd stay up all night ‘cause I'm afraid I might wet the bed and then my friends might make fun of me. (P3, 10‐year‐old girl)

The children expressed concerns about their academic performance, feeling unable to control it due to frequent hospitalisations and school absences. Participating in sports and physical activities, something enjoyed by most children and adolescents, is often not possible for those with TCS. They often find themselves excluded, observing from the sidelines as their peers engage in activities in playgrounds.

During gym class, I can only wander around, watching everyone else playing sports, chit‐chatting and having a blast. I know it's for my own good, but I can't help feeling like I'm on the outside looking in. (P9, 13‐year‐old girl)

#### 3.3.2 Subtheme 2: Familiar stranger

Many affected children avoid social engagement due to fear of stigma from odorous symptoms, often reinforced by instances of leakage, which makes them doubt their ability to form close friendships. Upon returning to school, they noticed a gradual distancing from former close friends, perceiving familiar gestures of greetings and assistance as more formal and charitable rather than genuinely friendly.

Even though my buddies have always looked out for me ‘cause of my sickness, like when I'm slow on the stairs, they lend a hand, and it feels nice. But they don't really chat with me anymore, not the close, heart‐to‐heart type stuff. It makes me wonder if they're not as close to me anymore because of my sickness. (P7, 11‐year‐old boy)

### 3.4 Theme 4: Striving for independence and consistency

Children often showed limited self‐care abilities, relying heavily on parents for daily needs. Yet, adolescents expressed shame about depending on parents for routine care, facing hurdles in becoming self‐sufficient. These obstacles to autonomy added to their emotional distress.

You know, I'm a big boy now, but my mom still treats me like I'm a tiny tot. And guess what? All she cares about is the poop problem I have, you know, when I never know when it's going to mess up my pants. Every time it happens, I just wanna disappear into a hole in the ground. I've tried, honest to goodness I have. I've tried to clean up the mess myself and to stop it from happening, but it's like I'm banging my head against a brick wall. Nothing seems to help. It's like I can't change this messy problem no matter what I do. (P10, 15‐year‐old boy)

Children often grapple with a desire for consistency, aiming to align their behaviour with their peers. Many children dealing with incontinence showed reluctance in carrying spare clothing or using diapers, highlighting their challenges in managing restroom visits.

You know, I just don't want to lug around extra clothes. I hate the thought of people knowing I have to change outfits. I just want to be like every other kid. Mom suggested that I wear diapers to school, but I shot that idea down real quick. It makes me feel super embarrassed. I'd rather go all the way home to change than stand out from everyone else. (P5, 12‐year‐old girl)

## 4 DISCUSSION

### 4.1 Main findings

This interview revealed various challenges postsurgery for paediatric TCS patients. Many experience persistent symptoms affecting daily life and development. They often perceive their condition as stigmatising, impacting personal growth, family communication and social interactions. This perceived stigma profoundly affects their emotional well‐being, leading to a sense of social unacceptability. Their language reflects intense emotional distress, frequently using terms like ‘shame’, ‘accident’, ‘terrible’ and ‘painful’ to describe their experiences, highlighting the emotional trauma linked to their condition.

### 4.2 What this study adds

Through the interview process, it was discerned that paediatric patients with TCS are confronted with substantial hindrances during their self‐development journey. Consistent with previous research, incontinence emerged as a paramount concern, often perceived as a deeply personal and stigmatising secret, consequently inducing a negative self‐image.^23^ The psychological burden associated with maintaining such a ‘secret’, particularly one that deviates significantly from societal norms, can have profound repercussions on the child's mental health. Existing literature substantiates the fact that the stigma linked to incontinence can precipitate social withdrawal, erode self‐esteem and escalate the risk of mental health disorders, such as anxiety and depression.^24^, ^25^ Our study observed varying responses among children with different functional levels and ages. Although gait instability and spinal curvature were present, they were not frequently discussed during interviews. Instead, incontinence emerged as the primary issue significantly affecting these children's mental state, persisting postsurgery, especially during the day. Adolescents, both entering puberty and already in it, faced challenges handling the shame linked to incontinence, intensifying emotional responses. This underscores the profound psychological impact of incontinence on paediatric TCS patients, overshadowing other concerns and significantly affecting their emotional well‐being. Recognising these emotional complexities across age groups and functional levels highlights the need for comprehensive interventions addressing the psychological toll of incontinence, alongside physical symptoms, especially during critical developmental stages. Moreover, their physical limitations often limit participation in activities, narrowing their view of abilities and potential. Research indicates these restrictions can hinder cognitive and emotional growth, impacting overall HRQoL.^26^, ^27^, ^28^ Concerns about education, careers and future paths create significant stress, affecting academic performance and future planning.^29^ Thus, the offering of tailored career guidance and the investigation of alternative educational methods could play a transformative role in framing their future outlooks.

During our interviews, we noticed significant challenges within familial relationships for children with TCS. They often face heightened parental concern and distrust due to their unique conditions, resulting in increased stress. The increased parental involvement can also strain family dynamics, affecting the child's autonomy and self‐assurance.^30^ This study reveals that children might perceive themselves as burdens to their families, leading to feelings of guilt. Research indicates this perception can lower self‐esteem and trigger depressive symptoms.^31^ Hence, it is crucial to introduce family counselling and education to prevent children from internalising these harmful perceptions.

The study highlighted significant social challenges faced by children with TCS. Their physical condition often leads to discrimination and exclusion in educational settings, fostering feelings of isolation and differential treatment in social interactions. Extensive research confirms these difficulties, emphasising their barriers to social integration, especially in traditional education.^32^ These challenges stem from peers' attitudes, limited understanding, physical constraints hindering full participation and experiences of isolation. Studies like Pinquart and Pfeiffer's^33^ show that children with physical disabilities are at a higher risk of loneliness, impacting their mental health, leading to increased depressive symptoms and reduced self‐esteem. To address this, it is crucial to develop strategies promoting social inclusion for children with TCS in educational and social environments.^34^

Additionally, the study illuminates how these children strive to blend in with peers and seek external validation by concealing their distinctive traits during social interactions, conforming to societal norms of a ‘typical’ child. This behaviour intensifies notably among adolescents, marked by personality maturation and increased social engagements. While this phase enhances psychological adaptability, it also heightens emotional volatility.^35^ How these children manage emotional regulation, friendships and self‐assessment can significantly shape their personality development.^36^ Notably, at this developmental stage, external perceptions hold significant weight against their self‐concept, potentially causing role confusion and impacting their psychological well‐being.^37^, ^38^ Peer comparison plays a pivotal role in shaping their self‐perception and behaviours, influencing not only their immediate psychological state but also their social adjustment and future paths. These insights provide a crucial understanding of the unique psychological landscape of these children, highlighting areas for focus in their educational and psychological support frameworks.

### 4.3 Implications of the study

Therefore, the study emphasises the need for a comprehensive postoperative care framework for children with TCS. This framework should prioritise their personal development, enhance family communication and foster social interaction skills. Such a holistic approach could facilitate their successful reintegration into both family and society, supporting a harmonious developmental path. Addressing the complex challenges these children encounter requires an integrated methodology beyond traditional medical interventions. A unified approach, incorporating psychological support, tailored learning strategies and engaging participatory methods, could drive their robust development. This highlights the significance of merging physical and mental healthcare in managing such conditions. Additionally, resolving intricate family issues demands a family‐centred model that improves communication, resolves conflicts and enhances parenting practices. By combining psychoeducation, therapy and social support, healthier family dynamics and overall child well‐being become attainable. Finally, strategies focusing on social integration in educational and societal settings should promote positive attitudes towards disabilities, improve accessibility to social activities and provide social skills training. These interventions have the potential to alleviate feelings of isolation and exclusion, thereby enhancing their societal well‐being and overall quality of life.

### 4.4 Limitations of the study

This investigation carries certain limitations that warrant acknowledgement. First, our use of purposive sampling introduces potential selection bias as it relies on the researcher's judgement, potentially aligning with preconceptions. Additionally, our study focused on children and adolescents reintegrating into society within a 3‐month period postsurgery, capturing primarily short‐term psychological states. Yet, these conditions often have enduring effects, warranting longer‐term psychological assessments for more comprehensive clinical insights. Moreover, the small sample size of 12 subjects, due to strict age criteria and geographical limitations, raises the risk of selection bias. Finally, conducting interviews solely in Mandarin and subsequent translation may introduce cultural biases, impacting the findings' generalisability. While relevant to similar contexts, caution is advised regarding their broader applicability.

## 5 CONCLUSION

This study explores the emotional journey of children with TCS as they reintegrate post‐surgery. Despite surgical intervention, they face ongoing challenges in development and relationships, distinct from their healthy peers. These difficulties, much like their unresolved symptoms, may persist long‐term. The findings empower healthcare providers, parents, educators and social workers with insights into these children's emotional experiences. This understanding can improve support systems, helping them shed feelings of shame and stigma, fostering a positive, inclusive environment for their unique childhood or adolescence.

## AUTHOR CONTRIBUTIONS

**Nan Lin**: Conceptualisation; methodology; software; investigation; validation; writing—original draft; writing—review and editing; formal analysis. **Mingxian Ren**: Conceptualisation; formal analysis; writing—review and editing. **Yujun Xiang**: Writing—review and editing; formal analysis. **Jiahuan Li**: Writing—review and editing; formal analysis. **Dan Wang**: Writing—review and editing; formal analysis. **Hongzhen Xu**: Writing—review and editing; resources; supervision; formal analysis.

## CONFLICT OF INTEREST STATEMENT

The authors declare no conflicts of interest.

## Supporting information

Supporting information.

## Data Availability

The data that support the findings of this study are available from the corresponding author upon reasonable request.
