# Supplementary material for: Exploring the life experiences of school‐aged children afflicted by tethered spinal cord syndrome: An interpretative qualitative study
Source: Health Expect. 2024 Jan 10;27(1):e13969. doi: 10.1111/hex.13969 (PMC10777609; doi:10.1111/hex.13969)
Supplement: Supplementary file 1 — Supporting information. [file HEX-27-e13969-s001.docx]

**Supplementary file**

**Topic Guide**

1. Have you encountered any challenges or periods of unhappiness since your discharge from the hospital, and how have you managed to overcome them?
2. Could you share some of the concerns or worries you've had?
3. How would you say your illness has impacted your life and academic progress?
4. Do you perceive any change in the way your family, classmates, or friends treat you now?
5. What type of assistance or support do you feel would be most beneficial for you at this time?
6. Can you describe your hopes or expectations for your future, including school, personal life, and other aspects?
